# Ca^2+^ mobilization-dependent reduction of the endoplasmic reticulum lumen is due to influx of cytosolic glutathione

**DOI:** 10.1186/s12915-020-0749-y

**Published:** 2020-02-26

**Authors:** Beáta Lizák, Julia Birk, Melinda Zana, Gergely Kosztyi, Denise V. Kratschmar, Alex Odermatt, Richard Zimmermann, Miklós Geiszt, Christian Appenzeller-Herzog, Gábor Bánhegyi

**Affiliations:** 1 Department of Medical Chemistry, Molecular Biology and Pathobiochemistry, Semmelweis University, Budapest, Hungary; 2 Division of Molecular and Systems Toxicology, Department of Pharmaceutical Sciences, University of Basel, Klingelbergstrasse 50, 4056 Basel, Switzerland; 3 Department of Physiology, Faculty of Medicine, Semmelweis University, Budapest, Hungary; 4 “Momentum” Peroxidase Enzyme Research Group of the Semmelweis University and the Hungarian Academy of Sciences, Budapest, Hungary; 5 Medical Biochemistry and Molecular Biology, Saarland University, 66421 Homburg, Germany; 6 University Medical Library, University of Basel, Spiegelgasse 5, 4051 Basel, Switzerland

**Keywords:** Endoplasmic reticulum, Endoplasmic reticulum stress, Redox homeostasis, Glutathione, Calcium, Sec61 translocon, Cyclosporine A, Cyclophilins, Calreticulin, Membrane transport proteins

## Abstract

**Background:**

The lumen of the endoplasmic reticulum (ER) acts as a cellular Ca^2+^ store and a site for oxidative protein folding, which is controlled by the reduced glutathione (GSH) and glutathione-disulfide (GSSG) redox pair. Although depletion of luminal Ca^2+^ from the ER provokes a rapid and reversible shift towards a more reducing poise in the ER, the underlying molecular basis remains unclear.

**Results:**

We found that Ca^2+^ mobilization-dependent ER luminal reduction was sensitive to inhibition of GSH synthesis or dilution of cytosolic GSH by selective permeabilization of the plasma membrane. A glutathione-centered mechanism was further indicated by increased ER luminal glutathione levels in response to Ca^2+^ efflux. Inducible reduction of the ER lumen by GSH flux was independent of the Ca^2+^-binding chaperone calreticulin, which has previously been implicated in this process. However, opening the translocon channel by puromycin or addition of cyclosporine A mimicked the GSH-related effect of Ca^2+^ mobilization. While the action of puromycin was ascribable to Ca^2+^ leakage from the ER, the mechanism of cyclosporine A-induced GSH flux was independent of calcineurin and cyclophilins A and B and remained unclear.

**Conclusions:**

Our data strongly suggest that ER influx of cytosolic GSH, rather than inhibition of local oxidoreductases, is responsible for the reductive shift upon Ca^2+^ mobilization. We postulate the existence of a Ca^2+^- and cyclosporine A-sensitive GSH transporter in the ER membrane. These findings have important implications for ER redox homeostasis under normal physiology and ER stress.

## Background

The lumen of the endoplasmic reticulum (ER) is the first compartment of the eukaryotic secretory pathway. Its content resembles that of an “extracellular space inside the cell.” For example, it is characterized by a high Ca^2+^ concentration and an oxidizing redox balance [1–3], whereas the term “redox balance” shall herein refer to the thiol/disulfide system only.

Proper maintenance of the intraluminal homeostasis in the ER is a vital requirement for the cell. Either the depletion of luminal Ca^2+^ or the alteration of the redox balance can lead to ER stress that is an ominous accumulation of unfolded proteins in the ER lumen. ER stress triggers an adaptive program of signal transduction pathways, called the Unfolded Protein Response (UPR). Unresolved ER stress can finally result in programmed cell death [4].

The ER lumen serves as the main source of releasable Ca^2+^ for cytosolic signaling, which is maintained by the Sarcoplasmic/Endoplasmic Reticulum Calcium ATP-ase (SERCA) pump. SERCA-dependent Ca^2+^ influx is counterbalanced by a basal Ca^2+^ leakage and the opening of various second messenger gated channels activated by different extracellular stimuli [5]. Besides being the Ca^2+^ store, the high luminal Ca^2+^ concentration is indispensable for the function of critical components of the protein folding machinery such as chaperones and folding enzymes [6, 7].

Formation of native disulfide bonds in secretory and membrane proteins is a crucial step in protein maturation. Oxidation of cysteine residues in nascent polypeptides or rearrangement of misplaced disulfide bonds is catalyzed by the members of the Protein Disulfide Isomerase (PDI) family, the reoxidation of which can happen through various pathways [8]. The reduced glutathione (GSH) and glutathione-disulfide (GSSG) redox pair is the major low molecular weight thiol-disulfide buffer in the ER lumen [9]. Both GSH and GSSG were shown to directly react with the active centers of PDIs [10]. Total glutathione concentration in the ER reaches millimolar ranges providing an exceptionally high buffering capacity against oxidizing or reducing imbalances [9, 11, 12].

Participating in second-order thiol-disulfide exchange reactions, the reducing power of glutathione depends on [GSH]^2^:[GSSG] rather than on the bimolecular ratio [GSH]:[GSSG] [9, 10]. The [GSH]^2^:[GSSG] ratio in the ER lumen is far more oxidizing than the cytosolic redox poise [13, 14]. This is also reflected by a higher [GSH]:[GSSG] [15]. The most recent estimation of ER luminal [GSH]:[GSSG] derives from intact HeLa cells using the glutathionylation state of a single cysteine mutant glutaredoxin, which calculated a bimolecular ratio of less than 7:1 [12]. According to these numbers, the ER luminal glutathione concentration [GSH]+2[GSSG] is twofold higher than the total cellular glutathione concentration [12].

The source of ER luminal GSH has to be the cytosolic glutathione pool, because the ER is devoid of enzymes for GSH synthesis. GSH was indeed shown to permeate the ER membrane; a facilitated diffusion selective to GSH was described in rat liver microsomes. On the other hand, microsomes were impermeable for GSSG, which was entrapped in the lumen upon GSH addition [16]. GSH permeation from the cytosol was also confirmed by showing the direct modification of luminal oxidoreductases by GSH [17, 18]. GSH can be directly oxidized by many intraluminal reactions involving the oxidative protein folding machinery; thus, the [GSH]:[GSSG] ratio is constantly shifted towards the oxidized form. The locally accumulated GSSG can leave the ER through the secretory pathway or can also react with reduced PDI for subsequent disulfide bond formation in client proteins [19, 20].

Buffering the luminal [GSH]^2^:[GSSG] ratio is indispensable for correct disulfide bond formation; therefore, it is strictly regulated by luminal oxidoreductases [21]. An over-oxidizing environment can lead to unwanted disulfide bond formation, which in turn can provoke the UPR or, in serious cases, apoptosis [22]. On the contrary, an over-reducing environment prevents disulfide bond formation and protein secretion; however, it can help the clearance of misfolded polypeptides. Since the maintenance of a proper redox distribution in PDIs active sites depends on the reducing power of GSH [17], the control of GSH uptake from the cytosol can be an important question.

Recently, several groups reported a reducing shift of the ER luminal redox balance upon Ca^2+^ depletion [13, 23, 24]. Inhibition of Ca^2+^ uptake by SERCA pump or hormones inducing ER Ca^2+^ release caused immediate reduction of the ER lumen. Biophysically different redox-sensitive fluorescent readouts like the fluorescent lifetime of roGFPiE [23] or the excitation ratio of Grx1-roGFP1-iE_ER_ [25] or an OxyR-YFP fusion protein called HyPer-ER [24] observed the same phenomenon in living cell experiments. Whereas roGFPiE reacts with thiol-disulfide couples [23] and HyPer-ER with thiol-disulfide couples or H_2_O_2_ with unclear specificities [26, 27], Grx1-roGFP1-iE_ER_ is a bona fide [GSH]^2^:[GSSG] sensor [13]. A direct Ca^2+^ sensitivity of the probes was also excluded [23], suggesting that indeed Ca^2+^ cues can physiologically regulate the ER redox balance. The rapid reductive shift can be explained by a quick change of the local concentrations of redox active compounds, either by the uptake of reducing or by the release of oxidizing molecules. Furthermore, rapid activation/inhibition of ER oxidoreductases upon Ca^2+^ depletion might also influence the luminal redox balance. In this vein, it was supposed that selective sequestration of PDI1A with calreticulin (CRT) in a complex which formed under Ca^2+^-depleted conditions decreases the effective concentration of this major thiol oxidant, resulting in a hypo-oxidizing state [28]. The same study also showed that the major ER thiol oxidase ERO1 was insensitive to changes in [Ca^2+^] [28].

Ca^2+^ mobilization also triggers a rapid increase in [ATP] in the ER lumen [29]. The underlying mechanism involves the ER membrane ATP/ADP exchanger AXER, which increases ATP import following enhanced glycolytic flux downstream of a Ca^2+^-dependent CAMKK2-AMPK signaling cascade [30], and a likely temporary lowering of ATP consumption in the ER in response to ER Ca^2+^ depletion [31]. Still, the molecular identification of most of the transporter proteins in the ER membrane is still missing, although biochemical evidence describing many carrier-mediated transport processes is available [32]. Non-specific membrane permeation possibilities also exist, for example, the translocon polypeptide channel was described as a pore in the ER membrane allowing the transition of ions including Ca^2+^, and several small molecules [33, 34]. The permeability of the translocon pore is known to be regulated by BiP, the most prominent chaperone of the ER lumen being a Ca^2+^-binding protein itself [35]. A recent study reported that the translocon in yeast can mediate GSH influx into the ER and that the channel is gated by oxidized Kar2, the yeast orthologue of BiP [36].

In this study, we further examined the mechanism of Ca^2+^-sensitive reduction of the ER lumen by real-time measurements using Grx1-roGFP1-iE_ER_ and HyPer-ER and found evidence for a Ca^2+^ depletion-driven GSH transport process through the ER membrane.

## Results

### Reduction of the ER lumen triggered by ER Ca^2+^ depletion depends on cellular glutathione

Recent studies using fluorescent redox sensors targeted into the ER revealed that depletion of the organelle’s Ca^2+^ store leads to a reductive shift in luminal redox balance in the time scale of minutes [13, 23, 24]. Either an irreversible inhibitor of SERCA (thapsigargin, TG) or the physiological Ca^2+^ mobilizing agents histamine [24] and cholecystokinin [23] rapidly transformed the ER luminal environment into a more reducing milieu. Although ER redox poise is known to control Ca^2+^ pumps and channels [37, 38], the relationship in the opposite direction, namely how Ca^2+^ can regulate the redox balance, has not been fully elucidated (for a recent review, see [39, 40]).

Given the close redox links between ER and mitochondria [41, 42], we initially evaluated the possibility of a mechanism that involves the mitochondria. However, neither mitochondrial superoxide production nor mitochondrial membrane potential or respiration was conspicuously affected by short-term (5–15 min) treatment with TG (Additional file 1: Fig. S1). What is more, ER stress over a longer time period leads to regulated protein reflux to the cytosol in budding yeast [43, 44]. Here, however, short-term ER Ca^2+^ depletion was not associated with the relocalization of the fluorescent redox sensor to the cytosol, as evidenced by co-staining immunofluorescence microscopy (Additional file 2: Fig. S2).

**Fig. 1:**
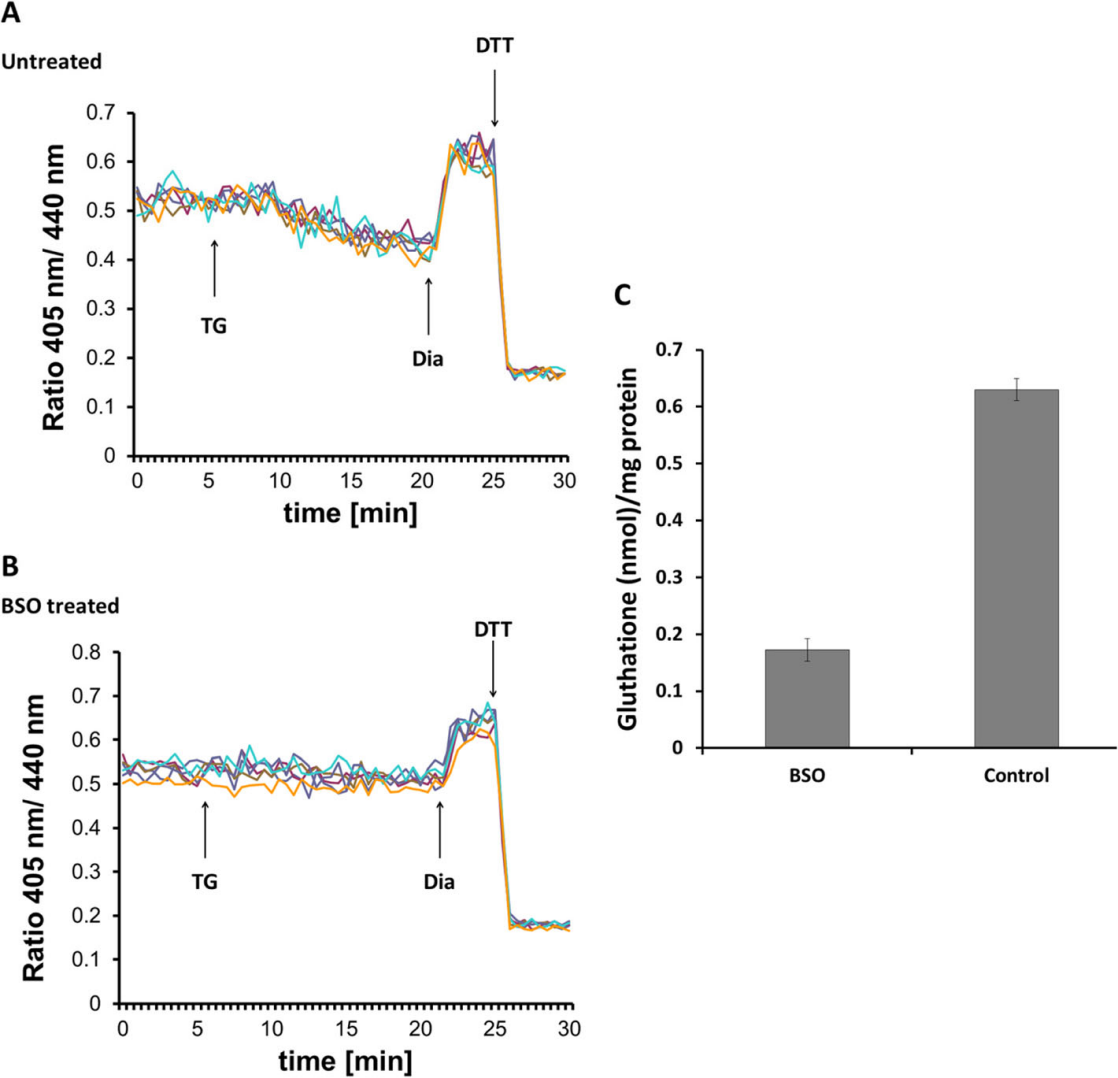
Ca^2+^ depletion-triggered ER reduction is sensitive to glutathione depletion by BSO. HEK293 cells were stably transfected with Grx1-roGFP1-iE_ER_ constructs and subjected to ratiometric laser scanning microscopy on a temperature-controlled stage with CO_2_ control. Fluorescence ratio changes were monitored over time. Each trace corresponds to the data recorded from one cell; traces were obtained from two independent experiments. One micromolar TG were applied to untreated (**a**) or BSO-treated (**b**) cells as indicated by the arrow. At the end of each experiment, 500 μM diamide (Dia) and 20 mM DTT were added to ensure the functionality of the probe. **c** Determination of total glutathione concentration by glutathione reductase assay as described in the “Materials and methods” section. One millimolar BSO treatment was performed overnight prior to experiment

We further reasoned that such rapid change in redox poise could plausibly be explained by induced influx of reductants from the cytosol or efflux of oxidants to the cytosol. Because ER luminal redox poise strongly depends on the [GSH]^2^:[GSSG] ratio, we first measured how the Ca^2+^ depletion-induced reductive shift was influenced by cellular glutathione levels. For monitoring ER redox poise, we used HEK293 cells stably expressing the specific [GSH]^2^:[GSSG] sensor Grx1-roGFP1-iE_ER_ [13]. Ratiometric measurements revealed that inhibition of SERCA by TG provoked a rapid reductive transition in Grx1-roGFP1-iE_ER_ redox state in agreement with previous results (Fig. 1a). However, when cellular GSH levels were depleted by overnight treatment with buthionine sulfoximine (BSO), the reductive transition upon addition of TG was abolished (Fig. 1b). BSO treatment resulted in a 75% drop of total glutathione concentration in HEK293 cells (Fig. 1c). We concluded each experiment by the consecutive addition of diamide and DTT to ensure the functionality of the probe. These results suggested that Ca^2+^ depletion-provoked reduction of the ER requires the cellular glutathione pool and residual glutathione in BSO-treated cells cannot mediate this process.

### Cytosolic Grx-roGFP2 is not detectably disturbed upon Ca^2+^ release

**Fig. 2:**
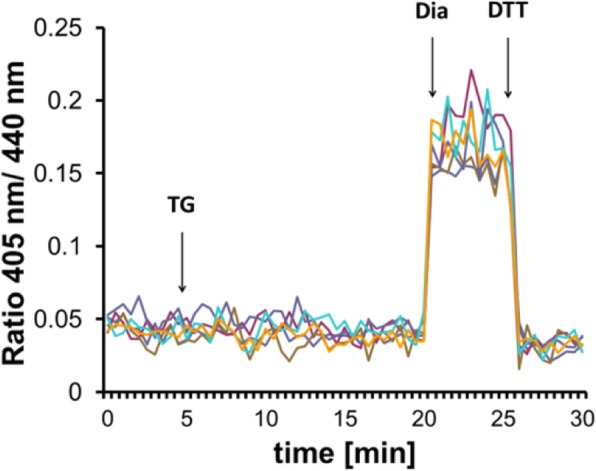
Cytosolic redox sensor Grx1-roGFP2 is not detectably disturbed upon thapsigargin-induced Ca^2+^ release. Fluorescence ratio changes of cytosolic Grx1-roGFP2 transiently expressed in HEK293. Traces correspond to data recorded from one cell; traces were obtained from two independent experiments. Cells were pretreated for 3 h before imaging with 100 μM of the GR inhibitor carmustine (BCNU) to prevent GSSG re-reduction. One micromolar TG were applied to cells as indicated by the arrow. At the end of the experiment, 500 μM diamide (Dia) and 20 mM DTT were added to ensure the functionality of the probe

In the ER lumen, the GSH/GSSG redox couple is shifted towards its oxidized form as a result of oxidative protein folding and the restricted permeability of GSSG through the ER membrane [16]. Accordingly, the quick reductive shift in response to TG could be caused by the flux of ER luminal GSSG to the cytosol, which would be expected to affect the cytosolic [GSH]^2^:[GSSG] ratio. This possibility was tested by monitoring the redox state of the cytosolic [GSH]^2^:[GSSG] sensor Grx1-roGFP2 [14] upon ER Ca^2+^ depletion. To prevent GSSG re-reduction by glutathione reductase (GR), Grx1-roGFP2-expressing HEK293 cells were pretreated with the GR-inhibitor carmustine (BCNU). We found that the redox poise of the cytosol was not measurably disturbed in response to TG (Fig. 2), suggesting that a mechanism other than GSSG efflux was responsible for the glutathione-dependent ER reduction.

### Permeabilization of the plasma membrane prevents the thapsigargin-induced reduction of the ER lumen

**Fig. 3:**
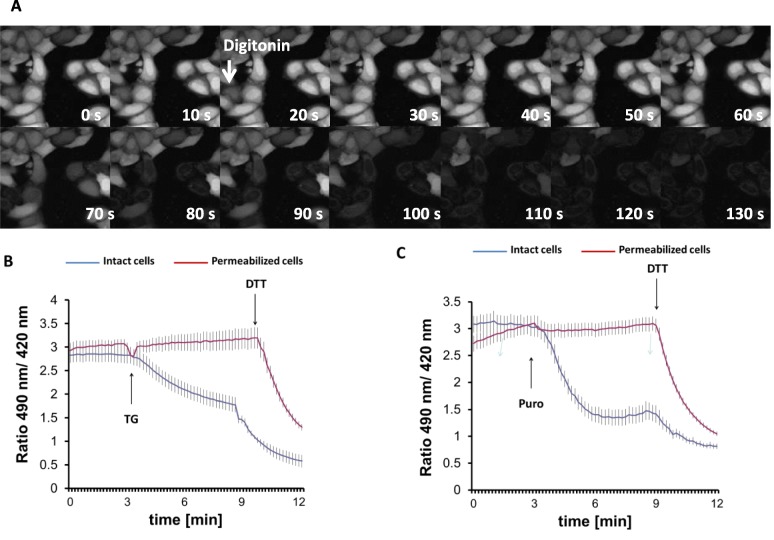
Permeabilization of the plasma membrane prevents thapsigargin-induced ER lumen reduction. **a** Sequential images of digitonin (25 μg/ml)-treated HeLa cells loaded with BCECF-AM fluorescent dye. **b**, **c** Fluorescence ratio changes of HyPer-ER sensor 24 h after transfection in digitonin-permeabilized (red line) or intact (blue line) HeLa cells. Cells were pretreated with digitonin for 3 min and washed with intracellular medium as described in the “Materials and methods” section prior to the experiment. TG (200 nM, **b**) or puromycin (100 μM, **c**) were applied at 3 min of imaging as indicated by the arrow. Experiments were terminated by addition of 0.5 mM DTT. Traces represent average intensity ratios acquired from 14 to 34 cells of 4 independent experiments

To further evaluate the possibilities of TG-induced ER import or export, we reasoned that global depletion of cytosolic components would affect the former possibility only. Digitonin selectively permeabilizes the plasma membrane due to its different lipid composition, but leaves intracellular membranes intact. Such treatment strongly dilutes the components of the cytosol and permits the examination of ER redox balance without cytosolic influence [18, 45]. The process of permeabilization was first visualized by monitoring the fluorescence decline in HeLa cells that were preloaded with BCECF-AM fluorescent dye (Fig. 3a). The persistence of the ER-localized fraction of the dye after 2 min of incubation with digitonin indicated the preserved integrity of the ER membrane (Fig. 3a). Using these optimized permeabilization conditions, HeLa cells were then transfected with HyPer-ER, permeabilized, and subjected to fluorescence ratio imaging. In this complex setup, we chose to use HyPer-ER rather than Grx1-roGFP1-iE_ER_ because of its superior dynamic range [46]. It is important to emphasize that this non-specific redox sensor reliably monitors the process of TG-induced ER reduction [24] (Additional file 3: Fig. S3). Digitonin did not seem to influence the steady-state redox state of HyPer-ER but abolished the TG-induced luminal reduction (Fig. 3b). This observation suggested that the rapid hypo-oxidation is strongly dependent on a cytosolic component such as GSH and disqualified the speculated efflux of oxidizing molecules such as GSSG.

### Thapsigargin increases glutathione levels in the ER lumen

**Fig. 4:**
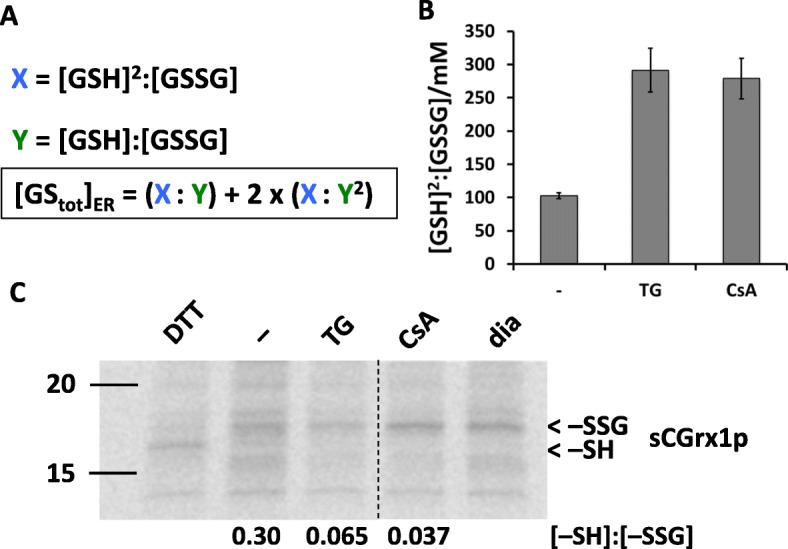
Thapsigargin and cyclosporine A increase glutathione levels in the ER lumen. **a** Formula for the calculation of [GS_tot_]_ER_ from [GSH]^2^:[GSSG] and [GSH]:[GSSG] in the ER. **b** [GSH]^2^:[GSSG] was quantified in the ER of Grx1-roGFP1-iE_ER_-expressing HEK293 cells that were left untreated (−) or treated with TG or CsA for 15 min by measuring the ratiometric emission intensity values of Grx1-roGFP1-iE_ER_ at steady state, fully oxidized, and fully reduced conditions. **c** sCGrx1p_ER_-transfected HEK293 cells were left untreated (−) or treated with TG or CsA for 15 min. Glutationylation state ([−SH]:[−SSG]) of sCGrx1p was determined by immunoprecipitation and TMMPEG modification of the radiolabelled protein. [−SH]:[−SSG] was quantified by SDS-PAGE, phosphor imaging, and densitometric analysis. Samples obtained from cells that were treated with 10 mM DTT or 5 mM diamide (dia) served as mobility markers for −SH and −SSG, respectively. The vertical dashed line indicates where an intervening lane has been removed. Note that [GSH]:[GSSG] is directly proportional to [−SH]:[−SSG]. One of three representative experiments is shown

We next analyzed possible changes in glutathione levels in the ER ([GS_tot_]_ER_). To this end, we used a recently published method for calculation of [GS_tot_]_ER_ [12] that combines the experimental values of [GSH]^2^:[GSSG] and [GSH]:[GSSG] (Fig. 4a). Thus, we first determined [GSH]^2^:[GSSG] in the ER by subjecting the Grx1-roGFP1-iE_ER_-expressing HEK293 line to a quantitative plate-reader assay [25] before and after treatment with TG for 15 min. Consistent with the results above, ER [GSH]^2^:[GSSG] rose from 103 ± 4 to 291 ± 33 mM upon treatment with TG (Fig. 4b). To determine ER [GSH]:[GSSG], we transfected HEK293 cells with ER-targeted sCGrx1p [12], which specifically equilibrates with [GSH]:[GSSG] [47]. As shown in Fig. 4c, the reduced sCGrx1p:glutathionylated sCGrx1p ratio ([−SH]:[−SSG]) did not parallel the reductive shift of [GSH]^2^:[GSSG] upon TG. In fact, [−SH]:[−SSG] rather decreased in response to treatment with TG. [−SH]:[−SSG] is proportional to [GSH]:[GSSG] [12] but, in the range below 0.1, can only be approximated by densitometry. We therefore only qualitatively concluded that ER [GSH]:[GSSG] remains constant or decreases in response to TG and that—according to the formula in Fig. 4a—[GS_tot_]_ER_ increases concomitantly to Ca^2+^ depletion-induced reduction of the ER. Together with the above results using BSO, Grx1-roGFP2, and digitonin, these data strongly indicated that cytosolic GSH enters the ER lumen upon ER luminal Ca^2+^ depletion.

### Chelation of cytosolic Ca^2+^ does not inhibit glutathione transport

**Fig. 5:**
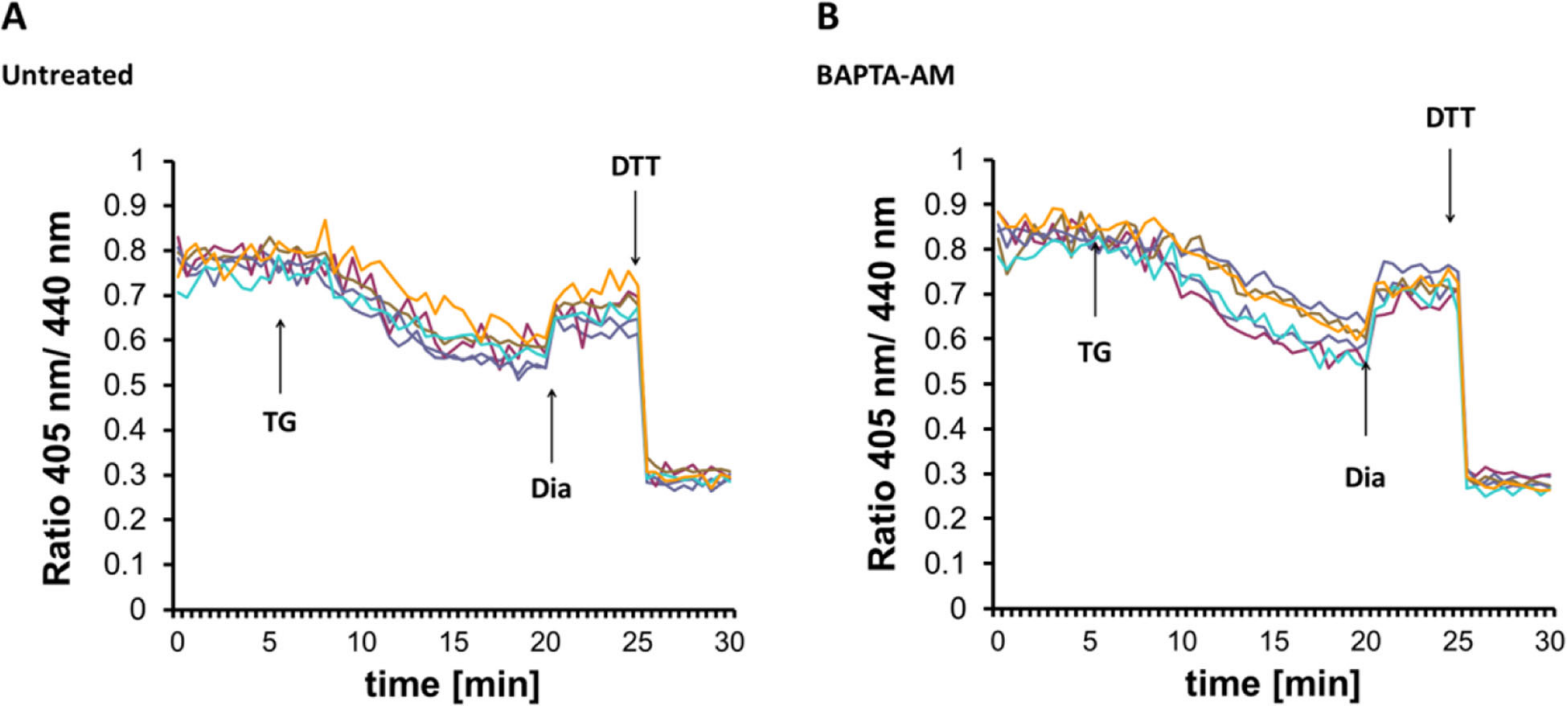
Chelation of cytosolic Ca^2+^ does not inhibit glutathione transport. Effect of 1 μM TG on the fluorescence ratio changes of Grx1-roGFP1-iE_ER_ in HEK293 cells left untreated (**a**) or pretreated with the Ca^2+^ chelator BAPTA-AM (**b**). Each trace corresponds to the data recorded from one cell

The depletion of ER Ca^2+^ is always accompanied by an increase in the cytosolic Ca^2+^ concentration. To resolve which side plays a role in the regulation of GSH transport, we buffered cytosolic Ca^2+^ with the chelator molecule BAPTA. Irrespective of hampered cytosolic Ca^2+^ fluxes, TG provoked the prompt reduction of the luminal GSH sensor, indicating that the decrease in luminal rather than the increase in cytosolic Ca^2+^ content triggers GSH transport (Fig. 5). These observations are consistent with the findings by Avezov et al. [23].

### Cyclosporine A promotes GSH transport into the ER

Members of the cyclophilin family have been reported to be resident in the ER [48, 49]. They participate in the regulation of oxidative protein folding and ERAD [49, 50]. Moreover, their prototypic inhibitor cyclosporine A (CsA) causes an oxidative shift in cellular glutathione, presumably by increasing the oxidation state of the ER [49]. On this basis, we investigated if CsA treatment inhibits Ca^2+^ release-triggered GSH transport.

**Fig. 6:**
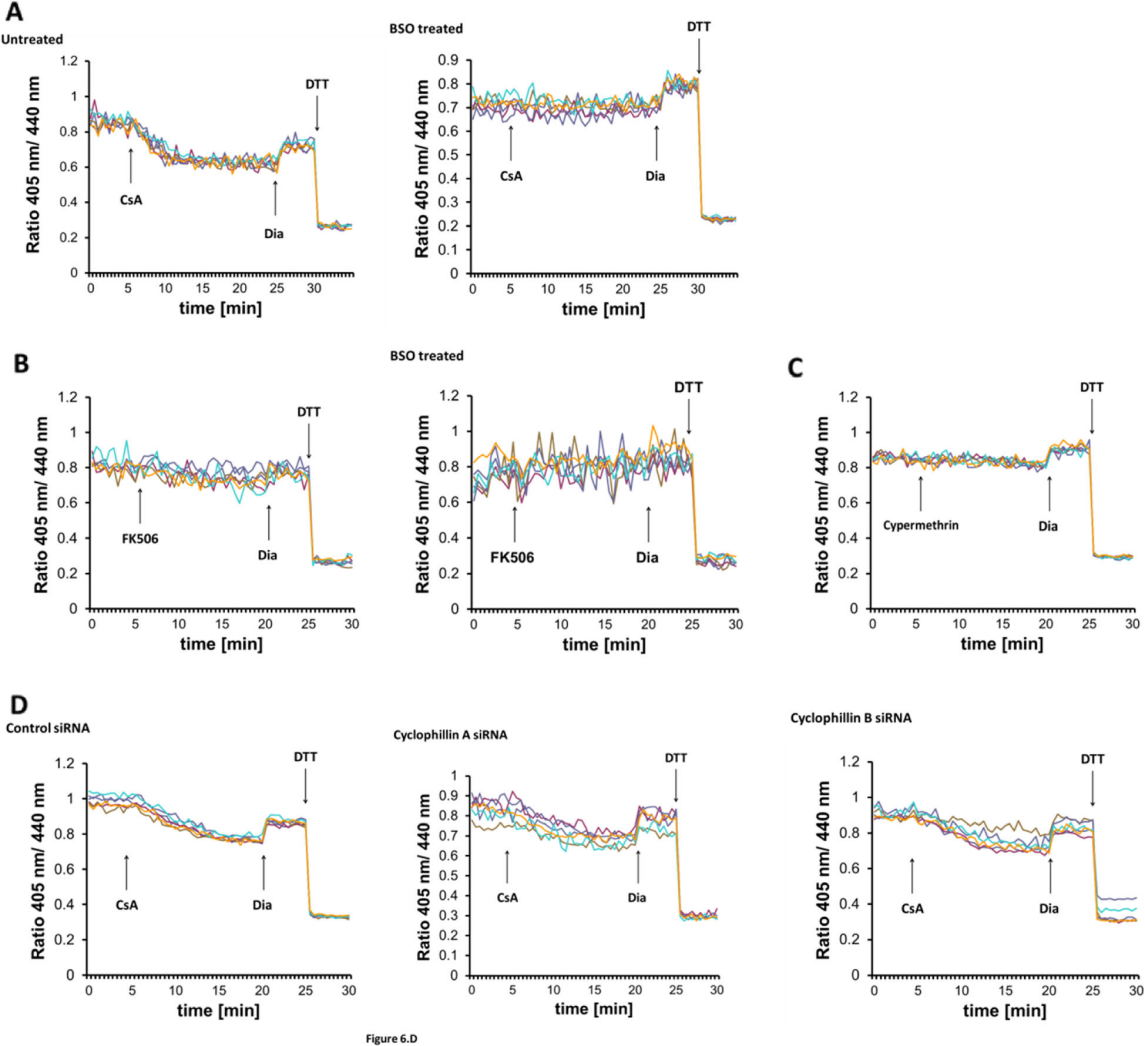
GSH transport can be triggered by cyclosporine A. **a** Real-time fluorescence ratio changes of Grx1-roGFP1-iE_ER_ in response to 10 micromolar CsA in HEK293 cells stably expressing the sensor. Each trace corresponds to the data recorded from one cell; traces were obtained from two independent experiments. At the end of each experiment, 500 μM diamide (Dia) and 20 mM DTT were added to ensure the functionality of the probe. Cells were left untreated or treated overnight with 1 mM BSO prior to the experiment. **b**, **c** Experiment performed as in **a**, but 50 μM FK506 (**b**) or 10 μM cyphermethrin (**c**) was applied as marked instead of CsA. **d** HEK293 cells stably expressing Grx1-roGFP1-iE_ER_ were transfected with control, cyclophillin A or B siRNA for 48 h before imaging; 10 μM CsA were applied as indicated by the arrow. Knockdown efficiency was verified by qPCR

Unexpectedly, real-time monitoring of Grx1-roGFP1-iE_ER_ revealed that CsA addition alone provoked the same immediate sensor reduction that was seen after TG addition (Fig. 6a). As for TG, the CsA-induced redox transition was sensitive to cellular GSH depletion (Fig. 6a) and short-term CsA treatment increased [GS_tot_]_ER_ (Fig. 4). Inhibition of both CsA- and TG-induced ER reduction by BSO strongly suggests a common, glutathione-centered mechanism.

The most thoroughly described mechanism of CsA action is the inhibition of the phosphatase activity of calcineurin, which prevents the activation of T lymphocytes [51]. CsA binds to the peptidyl-prolyl cis-trans isomerase cyclophilin A in the cytosol where the CsA-cyclophilin A complex mediates calcineurin inhibition [52]. To clarify, if calcineurin inhibition underlies ER reduction, we applied two mechanistically unrelated inhibitors of calcineurin, FK506 and cypermethrin [53]. Both calcineurin inhibitors failed to induce ER reduction, suggesting that the effect of CsA on ER GSH is independent of calcineurin (Fig. 6b, c). To test whether the GSH transporter might be directly gated by cyclophilins, we silenced the expression of cyclophilin A and the ER-resident cyclophilin B [48] and probed the sensor redox state after CsA addition. CsA-induced probe reduction was insensitive to the silencing of either cyclophilin (Fig. 6d), implying that CsA provokes GSH transport through another mechanism. Furthermore, although CsA is a well-known inhibitor of the mitochondrial permeability transition pore through blocking cyclophilin D [54], examination of its immediate effects on mitochondrial function showed only marginal changes (Additional file 1: Fig. S1).

### GSH transport into the ER is not mediated by Sec61

Glutathione is present in every cellular compartment [55]. However, although there are several reports on GSH transport through the ER membrane [16–18], an ER GSH transporter has not yet been identified [56].

**Fig. 7:**
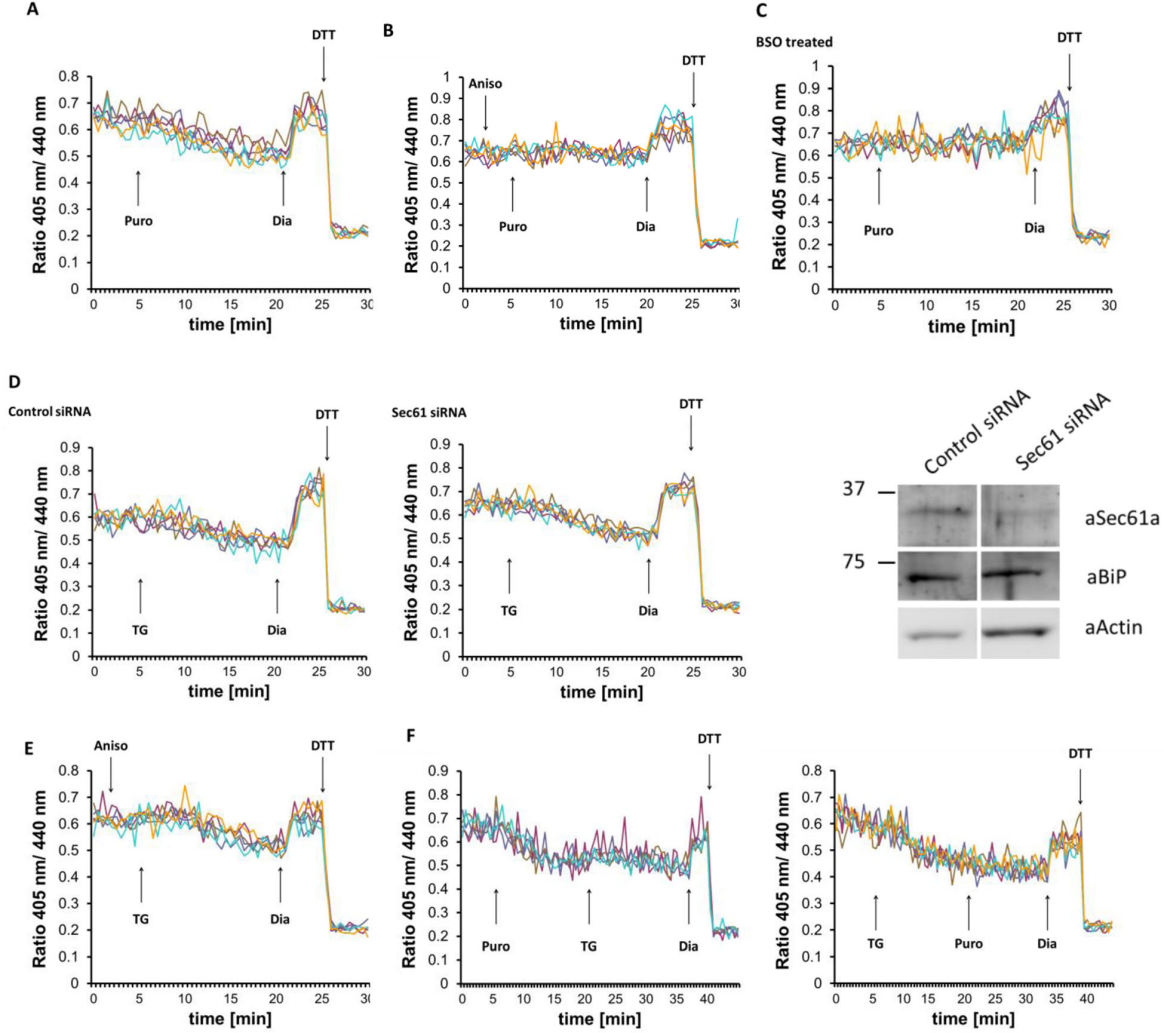
The Sec61 translocon polypeptide channel does not participate in glutathione transport. Effects of manipulating the translocon on fluorescence ratio changes of Grx1-roGFP1-iE_ER_ in HEK293 cells stably expressing the sensor. Each trace corresponds to the data recorded from one cell. At the end of each experiment, 500 μM diamide (Dia) and 20 mM DTT were added to ensure the functionality of the probe. **a** One hundred micromolar puromycin, **b** 200 μM anisomycin followed by 100 μM puromycin, **e** 200 μM anisomycin followed by 1 μM TG, and **f** 100 μM puromycin followed by 1 μM TG were applied as indicated by the arrow. **c** Cells were treated overnight with 1 mM BSO prior to experiment, and 100 μM puromycin were applied as marked. **d** HEK293 cells stably expressing Grx1-roGFP1-iE_ER_ were transfected with control or Sec61 siRNA for 48 h before imaging as above; 1 μM TG were applied as indicated by the arrow. Knockdown efficiency was verified by Western blot (aSec61a, anti-Sec61α antibody; aBiP, anti-BiP antibody; aActin, anti-actin antibody)

One possible candidate is the Sec61 translocon polypeptide channel, which allows the permeation of various small molecules through the ER membrane when not occupied by translocating polypeptide [34, 36]. Therefore, we examined whether opening the Sec61 channel would affect the ER luminal redox state. In agreement with earlier data [57], the application of puromycin, a translation inhibitor, which opens the Sec61 pore by clearing the nascent polypeptide, induced a comparable ER reduction as seen after TG addition (Fig. 7a). This reducing shift could be prevented by anisomycin (Fig. 7b), a known inhibitor of the unplugging action of puromycin [33]. Similar to TG, puromycin-induced ER reduction also depended on cellular glutathione levels, since BSO treatment or digitonin-mediated permeabilization of the plasma membrane abolished the reducing shift (Figs. 7c and 3c), but had no apparent effects on mitochondrial function (Additional file 1: Fig. S1).

The translocon channel can also act as a Ca^2+^ leak channel [58]. Therefore, opening of the translocon can either trigger Ca^2+^ release and indirectly induce Ca^2+^-sensitive GSH transport or directly facilitate the transport of GSH through the polypeptide channel itself. To distinguish between these two possibilities, we silenced Sec61 expression and examined the TG-induced redox change in Grx1-roGFP1-iE_ER_-expressing cells. Ca^2+^ depletion-dependent reduction was indistinguishable in Sec61-silenced and in non-silenced cells (Fig. 7d), suggesting that Sec61 was not directly involved in the transport of GSH.

We further examined whether Ca^2+^ depletion-induced reduction can be influenced by plugging the Sec61 translocon. Thus, cells were treated with anisomycin before TG addition. Since Sec61 is not the only possible Ca^2+^ leak channel in the ER membrane [34], we surmised that this treatment combination can further prove that Sec61 is dispensable for GSH transport. Indeed, sealing the Sec61 polypeptide channel with anisomycin did not prevent the TG-induced redox shift (Fig. 7e). We also applied puromycin before TG addition or in opposite order and observed no additive effect of the compounds in terms of hypo-oxidation of the ER lumen (Fig. 7f).

Kar2p, the yeast homolog of BiP, has recently been reported as a redox-dependent regulator of GSH influx into the ER through the Sec61 translocon [36]. Although our experiments in mammalian cells so far suggested that Sec61 was only indirectly involved in the inducible transport of GSH, we also checked for a possible regulation by BiP. However, neither the silencing of BiP nor its cleavage by subtilase toxin influenced the kinetics of ER reduction by CsA-induced GSH influx (Additional file 4: Fig. S4).

Together, this data argues that in mammalian cells, the Sec61 translocon does not participate in the Ca^2+^ depletion- or CsA-induced redox shift apart from serving as a Ca^2+^ leak channel in the presence of puromycin.

### Calreticulin is not required for the reduction of ER redox probes induced by Ca^2+^ depletion

Ca^2+^ depletion hampers the mobility of the ER oxidoreductase PDI1A in the ER, which was explained by complex formation at low [Ca^2+^] between the Ca^2+^-binding chaperone CRT and PDI1A [28]. Moreover, the TG-induced reductive shift, as measured by fluorescence lifetime of roGFPiE, appeared less prominent in CRT −/− mouse embryonic fibroblasts than in wild-type cells. It was concluded that the CRT-dependent decrease in mobility of PDI1A could be the mechanistic basis of Ca^2+^ depletion-induced ER hypo-oxidation [28].

**Fig. 8:**
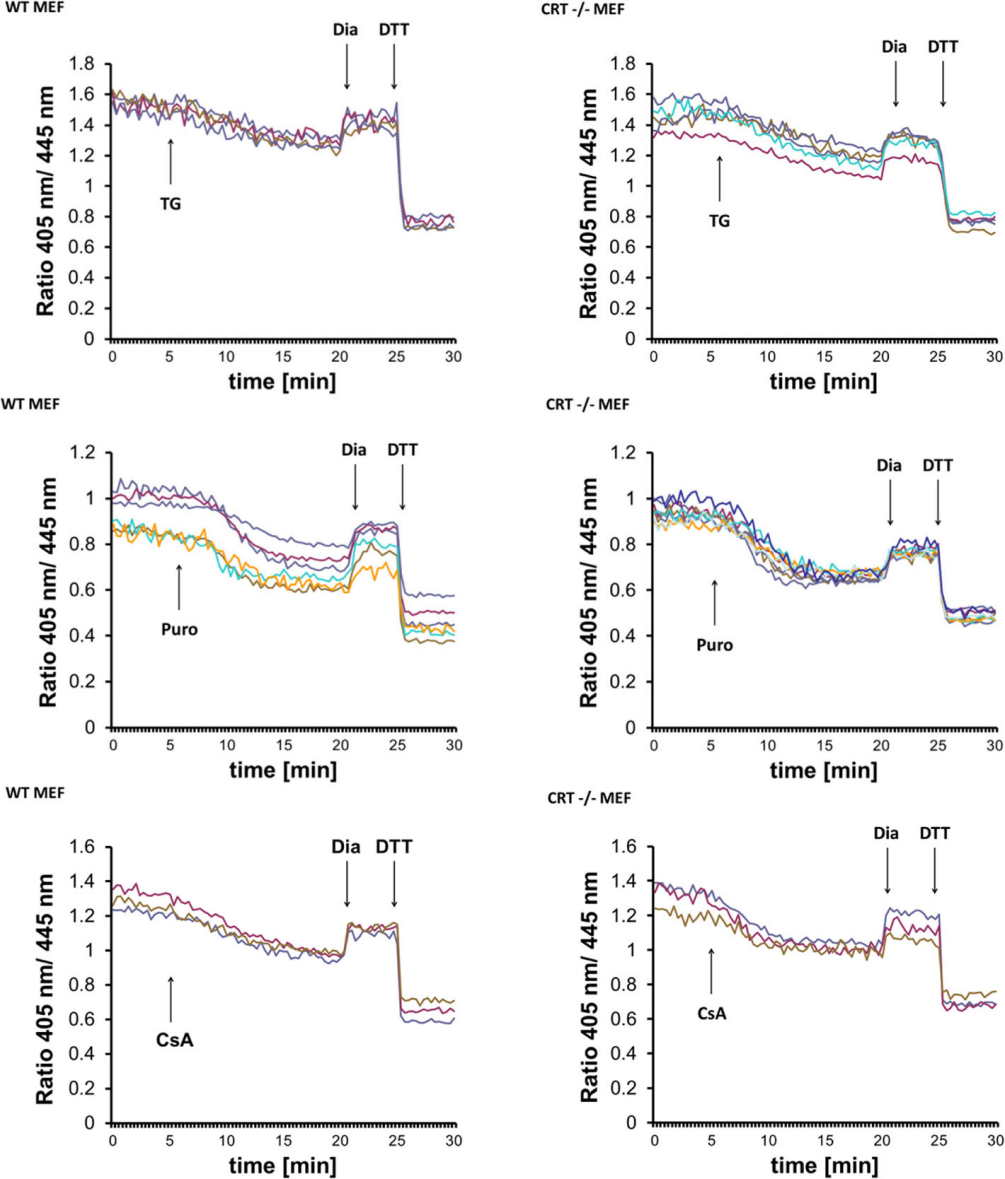
Calreticulin is dispensable for ER reduction induced by Ca^2+^ depletion or Cyclosporin A. Wild-type and CRT −/− mouse embryonic fibroblasts were transfected with Grx1-roGFP1-iE_ER_, and real-time fluorescent ratio changes were monitored. Reductive shift-provoking agents were applied as indicated. Each trace represents the data recorded from one cell; traces shown are representative of three independent experiments

In light of our new findings that inducible ER hypo-oxidation depends on bulk import of GSH from the cytosol, we revisited the CRT hypothesis in our system. To this end, wild-type and CRT −/− mouse embryonic fibroblasts were transfected with Grx1-roGFP1-iE_ER_ and analyzed by fluorescence video microscopy upon addition of TG, puromycin, or CsA. The fluorescence ratio curves of wild-type and CRT −/− cells equally responded at the time point of compound addition (Fig. 8), strongly indicating that CRT is dispensable for the induction of GSH-dependent ER reduction.

## Discussion

The maintenance of ER thiol-disulfide balance is of vital importance for the proper functioning of luminal processes, particularly the oxidative protein folding. Productive oxidative protein folding in the ER critically depends on the supply of disulfide reductants, which are required to resolve mispaired disulfide crosslinks in folding substrates [59, 60]. Currently, there is evidence for two cytosol-to-ER shuttling pathways for disulfide reductants: (i) a NADPH/thioredoxin reductase (TrxR)-dependent pathway [61] and (ii) a mechanism for GSH import into the ER [18, 62, 63]. In mammalian cells, both of these pathways are molecularly ill-defined, as is their presumable functional complementarity. It has recently been suggested that the TrxR-dependent pathway operates under non-stress conditions, whereas the GSH import pathway with its almost non-limited reducing capacity is mainly activated upon stress [59]. Indeed, housekeeping protein reduction events during oxidative protein folding do not require ER luminal GSH [64], whereas the millimolar GSH pool in the ER [12] is instrumental for the non-catalyzed elimination of increased ER H_2_O_2_ pools under stress [11].

Depletion of luminal Ca^2+^, either during normal cellular physiology or upon addition of pharmacological agents, provokes a rapid and reversible shift towards a more reducing redox state of the luminal [GSH]^2^:[GSSG] ratio [13, 23, 24]. Ca^2+^ depletion-induced ER redox alterations are relevant in physiological conditions associated with Ca^2+^ signaling, such as the response of pancreatic cells to secretagogues and neuronal activity. Moreover, ER stress leads to a general decrease in luminal [Ca^2+^] [41, 65], whereas the resulting rapid supply of thiols might help to dissolve stress-dependent protein aggregates and/or ER H_2_O_2_ accumulation [11]. In principle, this redox shift could be due to (i) a transient decrease in the activity of luminal oxidases and/or oxidoreductases, (ii) the induction of a hypothetical luminal reductase, (iii) a transmembrane influx/efflux of reductants/oxidants, or (iv) a combination of these events. In this study, we present convincing evidence for the third possibility.

We found that Ca^2+^ depletion-induced luminal reduction requires the presence of cytosolic GSH: inhibition of GSH synthesis by BSO or the release of cytosolic GSH by selective permeabilization of the plasma membrane prevented the redox shift upon Ca^2+^ release. These observations suggested that GSH influx rather than GSSG efflux is responsible for the phenomenon. This interpretation was corroborated by the findings that the cytosolic redox state was not measurably changed after Ca^2+^ release from the ER and that [GS_tot_]_ER_ was elevated rather than diminished in response to TG. Taken together, these results showed that GSH influx is the mechanism of Ca^2+^ depletion-induced luminal reduction. Of note, the steady-state sensor oxidation within the ER was not changed in BSO-treated cells. This is consistent with previous findings that the redox state of PDI-family members does not change in response to BSO-mediated glutathione depletion [19]. The subcellular distribution of glutathione in BSO-treated cells is currently unclear and warrants further research.

Ca^2+^ depletion also influences the mobility of PDIA1 via complex formation with the Ca^2+^-binding chaperone CRT [28]. PDIA1 is the major ER oxidoreductase that shuttles newly generated disulfides to a variety of disulfide acceptors such as nascent protein folding substrates and GSH [8]. CRT-dependent immobilization of PDIA1 at low [Ca^2+^] was proposed to explain the rapid ER reduction [28]. Consistently, the reductive shift of roGFP1iE that was induced by TG tended to be less prominent in CRT −/− mouse embryonic fibroblasts. This implies that in a Ca^2+^-depleted environment, the binding of PDIA1 to CRT slows down the rate of oxidative protein folding, thus provoking ER hypo-oxidation. However, given the ER glutathione concentration of several millimolar [12] and its immediate response within approximately 3 min [13, 23, 24], an explanation that argues with lowered input of newly generated disulfides appears insufficient for kinetic reasons. Indeed, our experiments with Grx1-roGFP1-iE_ER_ in wild-type and CRT −/− mouse embryonic fibroblasts showed equal responses to three reductive shift provoking agents, TG, puromycin, and CsA (Fig. 8). The discrepancy to the data from Avezov et al. could potentially be explained by the use of a glutathione-specific as opposed to a non-specific redox-sensing fluorescent protein reporter. The non-specific reporter used by Avezov et al. does not equilibrate with the glutathione redox couple [23] but may exhibit a certain selectivity to react with PDIA1 [66]. We conclude that CRT is dispensable for the rapid reduction of ER glutathione.

Glutathione biosynthesis resides exclusively in the cytosol [67], and glutathione transporters in intracellular membranes have not been identified at molecular level [55, 68]. However, functional studies revealed that GSH is able to cross the ER membrane, while permeation of GSSG is poor [16]. Since our work uncovers inducible GSH transport into the ER, we investigated the possible involvement of some candidate membrane proteins. The Sec61 translocon polypeptide channel has been reported to mediate the flux of some low molecular compounds beside proteins; however, the translocon opener puromycin did not enhance GSH transport significantly. A recent study postulated the translocon as an ER GSH transporter in yeast [36]. Indeed, the opening of the channel by puromycin reproduced the effect of TG on ER glutathione and the channel blocker anisomycin abolished the outcome, which also depended on the cytosolic GSH pool (Fig. 7a, b). However, silencing of Sec61 or plugging the channel with anisomycin did not result in the inhibition of TG-induced ER reduction. These results suggest that the Sec61 translocon behaves as one of several types of Ca^2+^ leakage channels in the ER but does not directly participate in inducible GSH transport through the ER membrane in human cells.

We previously proposed a model, by which the passive ER influx of cytosol-derived GSH followed by its oxidation to membrane-impermeable GSSG will “lower [GSH]_ER_ and set up a driving force for further import of GSH from the cytosol. According to this model, the ER would constitute a trap for cellular glutathione, which is reminiscent of the mechanism of osmosis where an impermeable metabolite drives the diffusion of a permeable metabolite across a selectivity barrier such as a biological membrane.” [12]. The finding that GSH can enter the ER by facilitated diffusion through the Sec61 translocon in a yeast mutant [36] is congruent with this model, even though it should be acknowledged that the toxic ~ 10-fold increase in cytosolic GSH in this yeast mutant represents a rather non-physiological situation with regard to glutathione gradients at intracellular membranes. The present data now rather suggests an active as opposed to passive GSH import mechanism across the ER membrane. We are still hesitant, however, to conclusively dismiss the possibility of passive GSH import along a cytosol-to-ER [GSH] gradient that may be maintained opposite to the ER-to-cytosol [GS_tot_] gradient reported earlier [12]. Such passive transport could be facilitated by a reversibly sealable, non-Sec61 permeation pore in the ER membrane.

We observed, much to our surprise, that CsA mimicked rather than inhibited the effect of TG on ER glutathione. Interestingly, cyclophilins, which are known targets of CsA, are involved in the regulation of the ER luminal milieu. On the one hand, overexpression of cytosolic cyclophilin A attenuates Ca^2+^ efflux from the ER, thereby inhibiting TG-induced apoptosis [69]. On the other hand, depletion of ER luminal cyclophilins results in ER hyper-oxidation with an elevated cellular GSSG:GSH ratio [49]. However, TG- and CsA-induced ER reduction was found to be independent of cyclophilins. The effect of CsA on ER glutathione was also independent of calcineurin, a prominent downstream target of the compound and a known modulator of ER Ca^2+^ channels [70]. CsA is also a prototypic inhibitor of glutathione- or glutathione conjugate-transporters of the ABC transporter superfamily, which operate in the plasma membrane [71]. However, our results showing a CsA-stimulated GSH influx into the ER speak against a possible involvement of ABC transporters.

Collectively, our data define a Ca^2+^- and CsA-sensitive transport mechanism of GSH at the ER membrane. This transport does not involve the translocon polypeptide channel or CsA-sensitive ABC transporters. We also excluded cyclophilins A and B, calcineurin, and CRT as regulatory components of GSH transport. Further studies are needed to explore this transport process in more detail.

## Conclusions

**Fig. 9:**
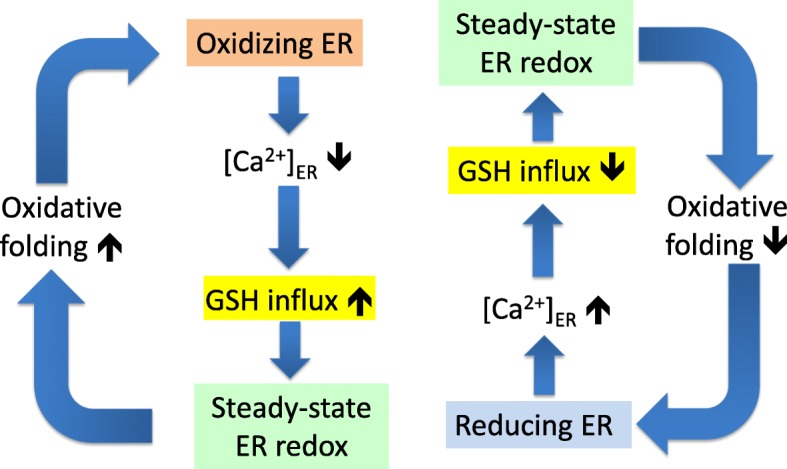
Schematic representation of feedback loops that connect ER Ca^2+^ loading, GSH influx, and oxidative protein folding. Hyper-oxidizing conditions in the ER (orange box) due to peak oxidative protein folding leads to Ca^2+^ depletion via opening of IP3R calcium channels and inhibition of SERCA pumps. Ca^2+^ depletion can in turn activate a GSH transporter (yellow box), which will restore the proper steady-state ER redox environment (green box). Conversely, hyper-reducing conditions in the ER (blue box) lower GSH influx via increased [Ca^2+^]_ER_, thereby rescuing steady-state ER redox and commensurate oxidative protein folding. These feedback mechanisms regulate the pace of oxidative protein folding and contribute to the robustness of ER luminal redox balance

Ca^2+^ mobilization from the ER results in influx of cytosolic GSH, which causes a redox shift towards more reducing conditions in the ER lumen. The mechanism may serve for the compensation of ER hyper-oxidation during excessive oxidative protein folding and/or ER stress. ER luminal redox-driven regulation of Ca^2+^ flux is well characterized and is known to involve inositol 1,4,5-trisphosphate receptors, ryanodine receptors, and sarco/endoplasmic reticulum Ca^2+^ transport ATPase [39, 40]. ER hyper-oxidation promotes Ca^2+^ release by the opening of ER Ca^2+^ channels and the inhibition of ER Ca^2+^ pumps. The present study unravels a homeostatic mechanism where Ca^2+^ depletion, in turn, can activate a GSH transporter, which will restore a proper ER redox environment (Fig. 9). This mechanism supports the feedback regulation of oxidative protein folding and contributes to the robustness of ER luminal redox balance.

## Materials and methods

### Generation of HEK293 cells stably expressing Grx1-roGFP1-iE_ER_

HEK293 cells were transfected with Grx1-roGFP1-iE_ER_/pcDNA3.1 [13] using Metafectene PRO (Biontex) and stably expressing clones selected by addition of 1 mg/ml G418 (Sigma). Homogeneous expression of clones was checked by fluorescence microscopy at the excitation wavelength of 405 nm. Clone D5 was selected for further experiments.

### Cell culture and transient transfections

HeLa and HEK293 cells were cultured in Dulbecco’s modified Eagle’s medium (DMEM) (Invitrogen) containing 4.5 g/l glucose supplemented with 10% fetal bovine serum, 100 U/ml penicillin, and 100 mg/ml streptomycin at 37 °C in 5% CO_2_. For cells stably expressing Grx1-roGFP1-iE_ER_, G418 (1 mg/ml) was added to the growth medium as a selection antibiotic.

Transient transfections with cytosolic Grx-roGFP2 [14] or HyPer-ER constructs [24] were performed with Lipofectamine (Thermo Fisher) reagent according to manufacturer’s instructions; cells were analyzed 48 h after transfection.

For silencing Sec61, HEK293 cells stably expressing Grx1-roGFP1-iE_ER_ were transfected using Lipofectamine RNAiMax reagent based on the manufacturer’s protocols, using final concentration of 100 nm of siRNA. Negative control and *SEC61A1* siRNA were previously published [58]. Successful knockdown was confirmed by Western blot analysis using anti-Sec61α primary antibody [58].

Silencing cyclophilin A and B siRNA was delivered by Lipofectamine RNAiMax (Thermo Fisher Scientific) according to manufacturer’s recommendation; 85 pM siRNA and 2.5 μl Lipofectamine reagent were used per 50,000 cells. The target sequence of the mock siRNA was 5′-UGGUUUACAUGUUUUCUGA-3′, of the cyclophilin A siRNA was 5′-CUGGAUUGCAGAGUUAAGU-3′, and of the cyclophilin B siRNA 5′-CAAAAACAGUGGAUAAUUU-3′ (Microsynth, Switzerland).

### Quantitative PCR and gene expression analysis

To assess gene expression, total RNA was extracted using TRI reagent (Sigma). Subsequently, cDNA was produced by reverse transcription with Maloney murine leukemia virus reverse transcriptase (Promega). Quantitative PCR (qPCR) analysis was performed using the KAPA SYBR Fast kit (Sigma) on a Rotor Gene Real-Time Cycler (Corbett Research). Normalization of the data relative to the endogenous control gene glyceraldehyde-3-phosphate dehydrogenase (GAPDH) was done according to the 2−ΔΔCt method for relative quantification.

Primers:

1. Human cyclophilin A

- FW: CAT CTG CAC TGC CAA GAC TGA
- Rev: TGC AAT CCA GCT AGG CAT G

2. Human cyclophilin B

- FW: GGT GAT CTT TGG TCT CTT CGG
- Rev: TAG ATG CTC TTT CCT CCT GTG

3. GAPDH

- FW: TGA TGA CAT CAA GAA GGT GGT GAA
- Rev: TCC TTG GAG GCC ATG TGG GCC AT

### Cultivation and transfection of mouse embryonic fibroblasts

The control and CRT −/− MEFs were kindly provided by Maurizio Molinari (Bellinzona, Switzerland) with the kind permission of Marek Michalak (Edmonton, Canada) [72]. The cells were cultured in αMEM containing 10% FBS and 100 U/ml penicillin and 0.1 mg/ml streptomycin under standard culture conditions (37 °C, 5% CO_2_). DNA transfection was performed using Xfect (Takara) according to manufacturer’s instructions; cells were analyzed 48 h after transfection.

### Live-cell imaging of Grx1-roGFP1-iE_ER_

Live-cell imaging was performed on an Olympus Fluoview 1000 (experiments for Fig. 8: Olympus Fluoview 3000) laser scanning confocal microscope equipped with a × 60 (experiments for Fig. 8, × 40) oil immersion objective (NA 1.40), a 405-nm laser diode, a-440 nm (experiments for Fig. 8, 445 nm) laser diode, and a 488-nm argon gas laser. The 405- and 440/445-nm laser lines were used as excitation wavelengths; the emission window was set to 500–600 nm. Images were acquired in sequential frame mode, separating the two channels. Grx1-roGFP1-iE_ER_-expressing cells were grown on glass bottom dishes (Mattek); for ratiometric analysis, cells were washed twice with DMEM without phenol red and transferred to a heated chamber (37 °C) with CO_2_ control. Reagents were added in 1 ml phenol red-free DMEM in the required concentration. For Ca^2+^ chelation experiments, cells were pretreated for 30 min with 50 μM BAPTA-AM. At the end of each experiment, 500 μM diamide and 20 mM DTT were added. Images were taken every 30 s for a period of 30 min and analyzed with the ImageJ software. One region of interest (ROI) per cell was chosen, which remained immobile for the duration of image acquisition, and 405/440 ratios were determined from emission intensities in background subtracted ROIs.

### Live-cell imaging of HyPer-ER

HeLa cells were analyzed 48 h after HyPer-ER transfection by fluorescent excitation ratiometry. Fluorescence-intensity measurements were performed on an inverted microscope (Axio Observer, Zeiss) equipped with a 40 × 1.4 oil-immersion objective (Fluar, Zeiss) and a Cascade II camera (Photometrics, Tucson, AZ). Excitation wavelengths were set by a random-access monochromator connected to a xenon arc lamp (DeltaRAM, Photon Technology International, Birmingham, NJ). For ratiometric measurements of HyPer-ER, excitation wavelengths of 490 and 420 nm were sequentially applied combined with a 505-nm dichroic filter and a 525/36-nm emission filter set. Cells grown on 10 cm coverslips were washed with HEPES-buffered solution containing 145 mM NaCl, 5 mM KCl, 1 mM MgCl_2_, 0.8 mM CaCl_2_, 10 mM HEPES, 5 mM glucose, and pH 7.4 and placed into a heated chamber at 37 °C. Reagents were added in 10× concentration in 0.1 ml of prewarmed buffer after removing 0.1 ml of medium. At the end of each experiment, 20 mM DTT was added to check sensor sensibility. Images were acquired every 10 s for a period of 30 min and analyzed by the MetaFluor (Molecular Devices, Downingtown, PA) software. Oxidation state of HyPer-ER was calculated by 490/420-nm fluorescence excitation ratio of HyPer-ER after background fluorescence subtraction.

For plasma membrane permeabilization, cells were treated with digitonin (25 μg/ml) for 3 min prior to experiment and washed with intracellular (IC) medium containing 113.5 mM KCL, 5 mM NaHCO_3_, 4 mM MgCl_2_, 40 nM CaCl_2_, 5 mM K-EGTA, 20 mM HEPES, 4 mM ATP, and 5.6 mM d-glucose. Experiments were performed after signal stabilization.

### Measurement of [GSH]^2^:[GSSG] and [GSH]:[GSSG] in the ER

To estimate [GS_tot_]_ER_, we used the procedure published in Montero et al. [12]. The degree of oxidation (OxD) of Grx1-roGFP1-iE_ER_ was quantitatively determined in cells stably expressing Grx1-roGFP1-iE_ER_ in 96-well plates (Falcon) in complete medium without phenol red. One day after seeding, cells were treated with 1 μM thapsigargin and 10 μM CsA, or left untreated for 15 min. The completely oxidized and reduced conditions were achieved by adding 500 μM diamide or 10 mM DTT to each pretreatment respectively before excitation spectrum analysis. Fluorescent intensities were measured on 520 nm from the bottom on Spectramax Gemini EM (Molecular Device) in a 350–500-nm range. OxD values and OxD-derived [GSH]^2^:[GSSG] values were calculated as published before [25].

The glutathionylation status of sCGrx1p_ER_ was analyzed in transiently transfected HEK293 cells by densitometric analysis of [^35^S]-methionine metabolically labeled, alkylated, and immunoprecipitated protein as described previously [12]. Cells were left untreated or treated with 1 μM thapsigargin or 10 μM CsA for 15 min ahead of analysis.

## Supplementary information

**Additional file 1: Figure S1.** Short-term thapsigargin, cyclosporine A and puromycin treatment has only minor effects on mitochondrial superoxide production, the mitochondrial membrane potential, and mitochondrial respiration. (A) Mitochondrial superoxide production was measured in HEK cells seeded at a density of 30000 cells per well to a 96 well plate the day before measurement. Cells were loaded with 5 μM MitoSOX red and Hoechst 33342 (both Life Technologies) for 10 minutes. The cells were treated with either 0.1% DMSO, 30% ethanol (positive control), 10 μM cyclosporine A (CsA), 1 μM thapsigargin, or 100 μM puromycin, and mitochondrial superoxide production was measured for the indicated times on a Cellomics ArrayScan VTI HCS Reader (Thermo Scientific). (B) Mitochondrial membrane potential was detected in HEK cells seeded at a density of 30000 cells per well to a 96 well plate the day before measurement. For positive control, cells were treated with 230 nM valinomycin (Sigma) for six hours. The cells were treated with either 0.1% DMSO, 10 μM cyclosporine A, 1 μM thapsigargin or 100 μM puromycin for 15 to 60 minutes in complete medium. The cells were stained for 30 minutes with Hoechst 33342 and MitoTracker Red CMXRos (both Life Technologies) and analyzed on a Cellomics ArrayScan VTI HCS Reader counting at least 1000 cells per well. Values were normalized to 15 minutes DMSO. (C) Left panel: Real-time measurements of oxygen consumption rate (OCR), reflecting mitochondrial respiration were performed on a Seahorse XF96 Analyzer (Agilent Technologies, USA) based on previous description (Nagy et al., Biochim Biophys Acta Bioenerg 2018, 1859, 201-214). Where indicated by arrows, cells were treated with metabolic inhibitors/modulators (oligomycin 2 μM, FCCP 100 nM, and antimycin A + rotenone 1 μM each). TG, puromycin, and CsA were applied 5 min ahead of the recording at the following concentrations: 1 μM, 100 μM, and 10 μM, respectively. Right panel: Basal respiration rate was detected before oligomycin addition; ATP-linked respiration was calculated as a difference between the values before oligomycin and before carbonyl cyanide-*4*-(trifluoromethoxy)phenylhydrazone (FCCP) addition; maximal respiratory capacity is the difference between values before and after rotenone + antimycin A addition; spare capacity difference between values before oligomycin and before rotenone + antimycin A addition; non-mitochondrial respiration is shown by the last value after rotenone + antimycin A addition. Data were obtained from two independent experiments (mean ± s.d). (PPTX 258 kb)

**Additional file 2: Figure S2.** Grx1-roGFP1-iE_ER_ is not released from the ER upon treatment of cells with thapsigargin, cyclosporine A, or puromycin for 30 min. HEK293 cells stably expressing HA-tagged Grx1-roGFP1-iE_ER_ were treated with 0.1 %DMSO, 10 μM cyclosporine A (CsA), 1 μM thapsigargin (TG) or 100 μM puromycin (Puro) for 30 min. Cells were fixed, stained with Hoechst 33342, incubated with anti-Calnexin and anti-HA antibodies followed by green- and red-fluorescent secondary antibodies, and analysed on an epifluorescence microscope. Overlay images including increased magnification frames of selected insets are shown for each treatment in colour. Size bar, 10 μm. (PPTX 2915 kb)

**Additional file 3: Figure S3.** BSO treatment prevents Hyper-ER reduction upon addition of thapsigargin. Fluorescence ratio changes of HyPer-ER sensor 24 hours after transfection in untreated or BSO treated HeLa cells. (PPTX 5979 kb)

**Additional file 4: Figure S4.** BiP does not influence the Ca^2+^ depletion-dependent reductive shift in the ER. Effect of manipulation of BiP on fluorescence ratio changes of Grx1-roGFP1-iE_ER_ in HEK293 cells stably expressing the sensor. BiP levels were diminished by the addition of subtilaseAB (SubAB) toxin or by silencing (BiP kd). As control, an inactive subtilaseA_A272_B mutant (SubAB mut) and a non-silencing control siRNA (control kd) were also used. The effect of subtilaseAB treatment (A) or BiP silencing (B) was checked by immunoblotting. (C) Identical curves of reductive shift were observed upon CsA addition in all cases; SubAB treatment for 60 min, siRNA transfection for 48 h. (PPTX 1031 kb)

## Data Availability

The datasets used and/or analyzed during the current study were deposited on Zenodo [73]. Reagents specific to this study are available on request.

## Abbreviations

BSO: Buthionine sulfoximine
CsA: Cyclosporine A
ER: Endoplasmic reticulum
TG: Thapsigargin
