# Supplementary material for: Ca2+ mobilization-dependent reduction of the endoplasmic reticulum lumen is due to influx of cytosolic glutathione
Source: BMC Biol. 2020 Feb 26;18:19. doi: 10.1186/s12915-020-0749-y (PMC7043043; doi:10.1186/s12915-020-0749-y)
Supplement: Supplementary file 1 — Additional file 1: Figure S1. Short-term thapsigargin, cyclosporine A and puromycin treatment has only minor effects on mitochondrial superoxide production, the mitochondrial membrane potential, and mitochondrial respiration. (A) Mitochondrial superoxide production was measured in HEK cells seeded at a density of 30000 cells per well to a 96 well plate the day before measurement. Cells were loaded with 5 μM MitoSOX red and Hoechst 33342 (both Life Technologies) for 10 minutes. The cells were treated with either 0.1% DMSO, 30% ethanol (positive control), 10 μM cyclosporine A (CsA), 1 μM thapsigargin, or 100 μM puromycin, and mitochondrial superoxide production was measured for the indicated times on a Cellomics ArrayScan VTI HCS Reader (Thermo Scientific). (B) Mitochondrial membrane potential was detected in HEK cells seeded at a density of 30000 cells per well to a 96 well plate the day before measurement. For positive control, cells were treated with 230 nM valinomycin (Sigma) for six hours. The cells were treated with either 0.1% DMSO, 10 μM cyclosporine A, 1 μM thapsigargin or 100 μM puromycin for 15 to 60 minutes in complete medium. The cells were stained for 30 minutes with Hoechst 33342 and MitoTracker Red CMXRos (both Life Technologies) and analyzed on a Cellomics ArrayScan VTI HCS Reader counting at least 1000 cells per well. Values were normalized to 15 minutes DMSO. (C) Left panel: Real-time measurements of oxygen consumption rate (OCR), reflecting mitochondrial respiration were performed on a Seahorse XF96 Analyzer (Agilent Technologies, USA) based on previous description (Nagy et al., Biochim Biophys Acta Bioenerg 2018, 1859, 201-214). Where indicated by arrows, cells were treated with metabolic inhibitors/modulators (oligomycin 2 μM, FCCP 100 nM, and antimycin A + rotenone 1 μM each). TG, puromycin, and CsA were applied 5 min ahead of the recording at the following concentrations: 1 μM, 100 μM, and 10 μM, respectively. Right panel: Basal respiration rat [file 12915_2020_749_MOESM1_ESM.pptx]

## Slide 1
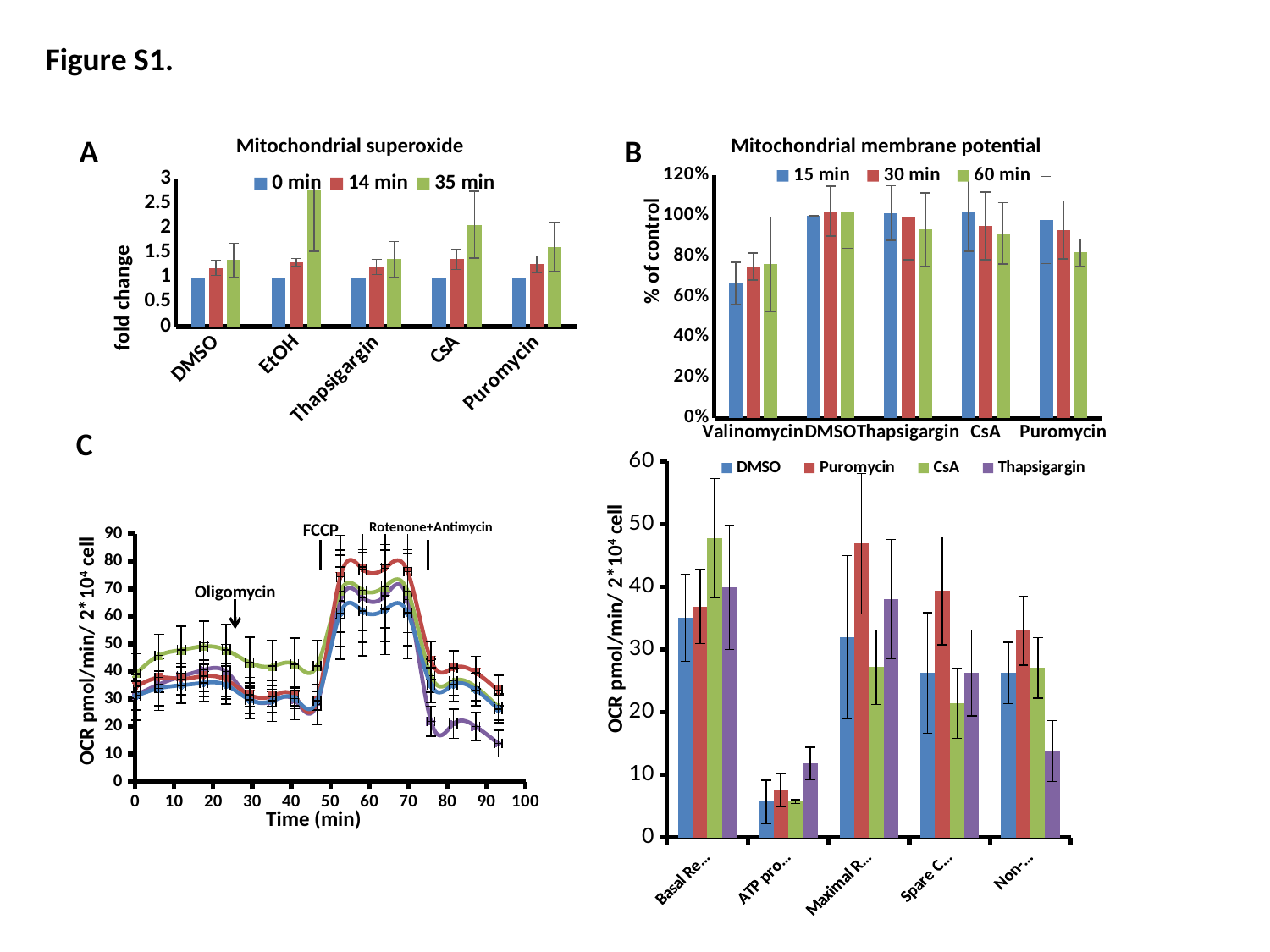

Figure S1.
A
Mitochondrial superoxide
B
Mitochondrial membrane potential
### Chart
| Category | 15 min | 30 min | 60 min |
|---|---|---|---|
| Valinomycin | 0.666035289594652 | 0.7497122068245782 | 0.7609259626788715 |
| DMSO | 1.0 | 1.0232978657479561 | 1.0227244288390223 |
| Thapsigargin | 1.0135809790083226 | 0.9968691990264359 | 0.9323702974752526 |
| CsA | 1.0218861694920052 | 0.9500856482133283 | 0.9146763287851583 |
| Puromycin | 0.9800720524539693 | 0.9307670399702701 | 0.8187060398301941 |
### Chart
| Category | | | |
|---|---|---|---|
| DMSO | 1.0 | 1.1868333972770457 | 1.3465032363949907 |
| EtOH | 1.0 | 1.2965642087694216 | 2.7609475947289783 |
| Thapsigargin | 1.0 | 1.2090987019854313 | 1.3626820005400344 |
| CsA | 1.0 | 1.360928488882131 | 2.0622702751621893 |
| Puromycin | 1.0 | 1.258191295949865 | 1.6109892698710742 |% of control
### Chart
| Category | DMSO | Puromycin | CsA | Thapsigargin |
|---|---|---|---|---|
| Basal Respiration | 35.05057843526204 | 36.88255373636882 | 47.75673548380534 | 39.91652997334798 |
| ATP production | 5.712954203287758 | 7.548377354939781 | 5.731653849283859 | 11.805754979451493 |
| Maximal Respiration | 31.99008560180664 | 46.9035987854004 | 27.18042755126953 | 38.05494499206543 |
| Spare Capacity | 26.277131398518883 | 39.35522143046062 | 21.448773701985672 | 26.249190012613937 |
| Non-Mito | 26.276430130004883 | 33.02707862854004 | 27.095799763997398 | 13.81718111038208 |OCR pmol/min/ 2*104 cell
C
FCCP
Rotenone+Antimycin
### Chart
| Category | DMSO | Puromycin | CsA | Thapsigargin |
|---|---|---|---|---|Oligomycin
OCR pmol/min/ 2*104 cell
Time (min)
