# Supplementary figures and images for: Ca2+ mobilization-dependent reduction of the endoplasmic reticulum lumen is due to influx of cytosolic glutathione
Source: BMC Biol. 2020 Feb 26;18:19. doi: 10.1186/s12915-020-0749-y (PMC7043043; doi:10.1186/s12915-020-0749-y)

## Slide 1
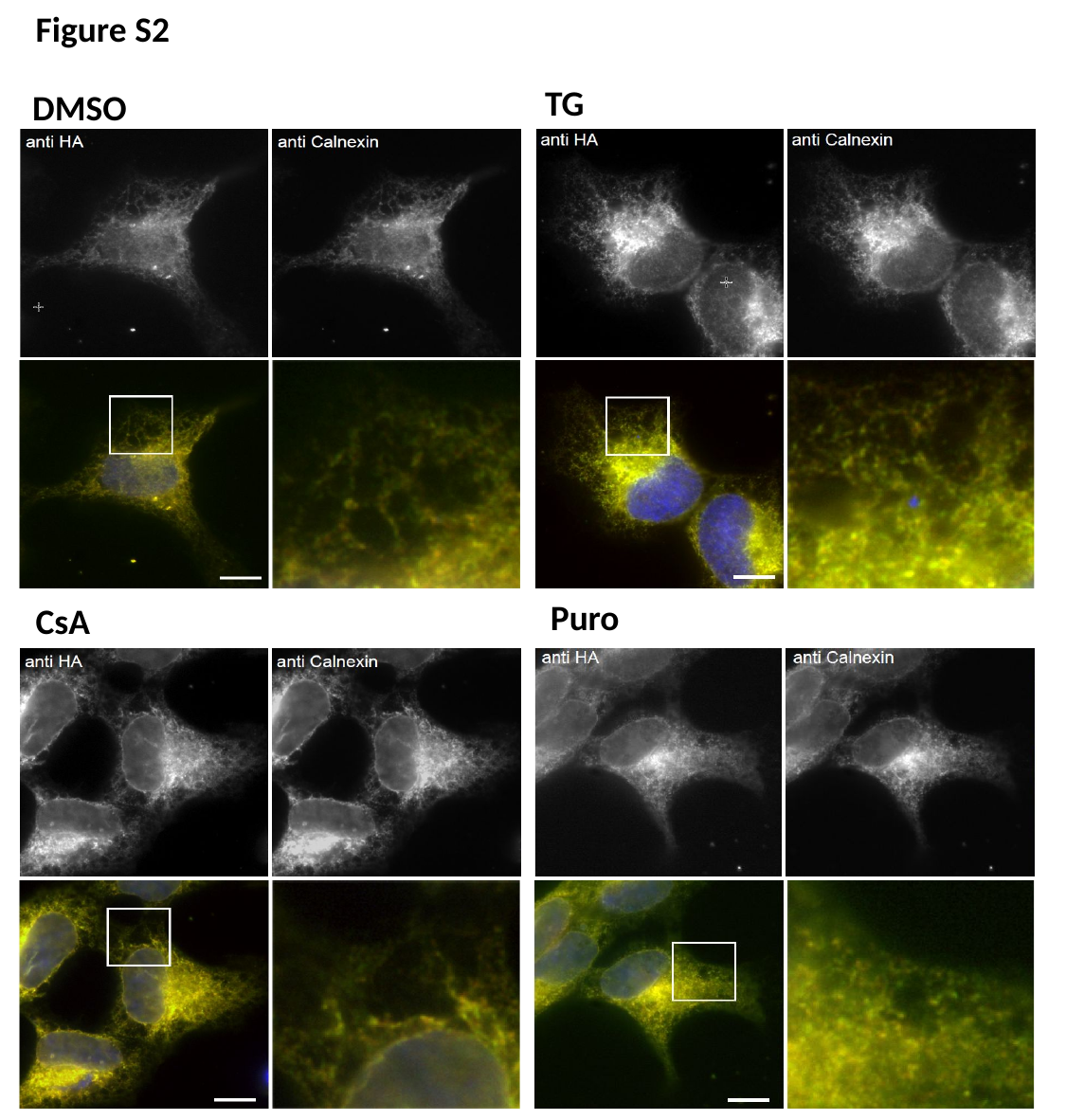

Figure S2
TG
DMSO
Puro
CsA

Supplement: Supplementary file 2 — Additional file 2: Figure S2. Grx1-roGFP1-iEER is not released from the ER upon treatment of cells with thapsigargin, cyclosporine A, or puromycin for 30 min. HEK293 cells stably expressing HA-tagged Grx1-roGFP1-iEER were treated with 0.1 %DMSO, 10 μM cyclosporine A (CsA), 1 μM thapsigargin (TG) or 100 μM puromycin (Puro) for 30 min. Cells were fixed, stained with Hoechst 33342, incubated with anti-Calnexin and anti-HA antibodies followed by green- and red-fluorescent secondary antibodies, and analysed on an epifluorescence microscope. Overlay images including increased magnification frames of selected insets are shown for each treatment in colour. Size bar, 10 μm. (PPTX 2915 kb) [file 12915_2020_749_MOESM2_ESM.pptx]

## Slide 1
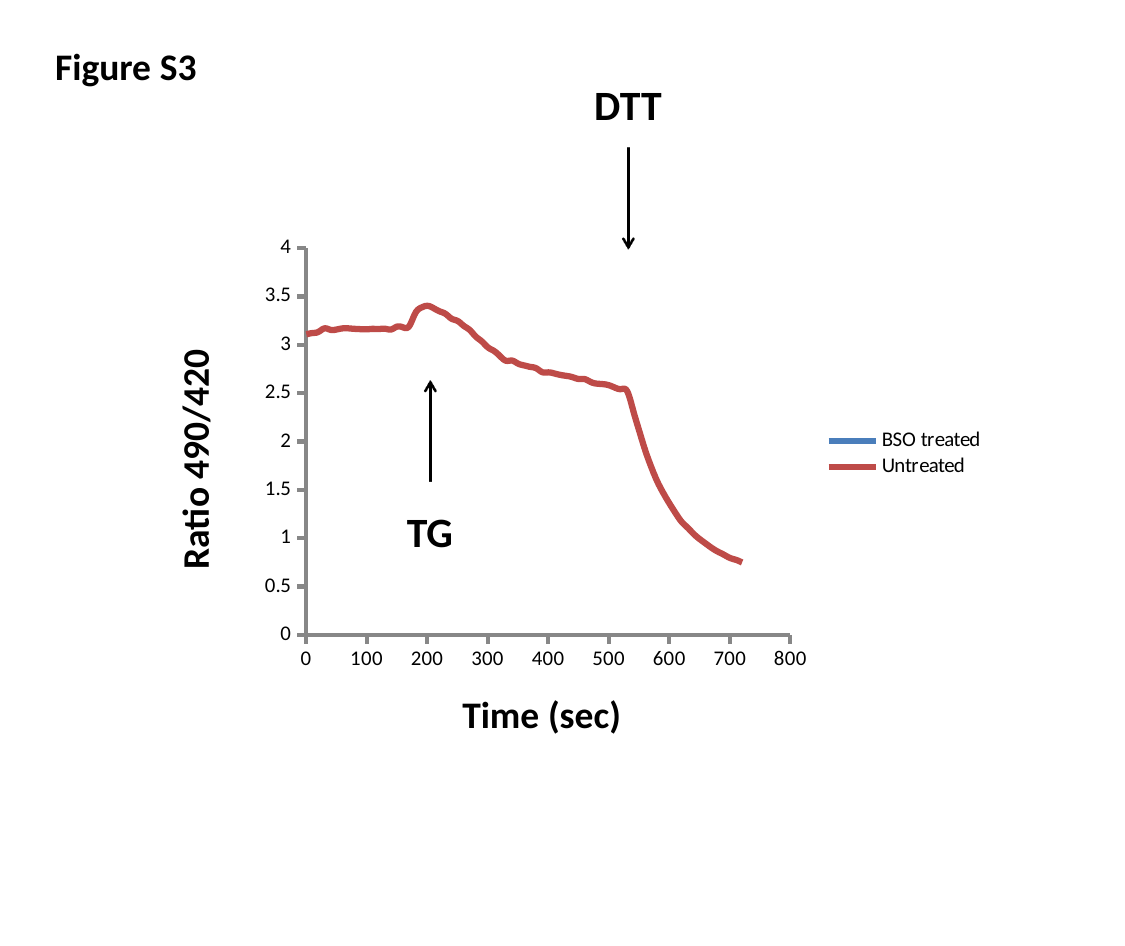

Figure S3
DTT
### Chart
| Category | BSO treated | Untreated |
|---|---|---|
Ratio 490/420
TG
Time (sec)

Supplement: Supplementary file 3 — Additional file 3: Figure S3. BSO treatment prevents Hyper-ER reduction upon addition of thapsigargin. Fluorescence ratio changes of HyPer-ER sensor 24 hours after transfection in untreated or BSO treated HeLa cells. (PPTX 5979 kb) [file 12915_2020_749_MOESM3_ESM.pptx]
