# Supplementary material for: Ca2+ mobilization-dependent reduction of the endoplasmic reticulum lumen is due to influx of cytosolic glutathione
Source: BMC Biol. 2020 Feb 26;18:19. doi: 10.1186/s12915-020-0749-y (PMC7043043; doi:10.1186/s12915-020-0749-y)
Supplement: Supplementary file 4 — Additional file 4: Figure S4. BiP does not influence the Ca2+ depletion-dependent reductive shift in the ER. Effect of manipulation of BiP on fluorescence ratio changes of Grx1-roGFP1-iEER in HEK293 cells stably expressing the sensor. BiP levels were diminished by the addition of subtilaseAB (SubAB) toxin or by silencing (BiP kd). As control, an inactive subtilaseAA272B mutant (SubAB mut) and a non-silencing control siRNA (control kd) were also used. The effect of subtilaseAB treatment (A) or BiP silencing (B) was checked by immunoblotting. (C) Identical curves of reductive shift were observed upon CsA addition in all cases; SubAB treatment for 60 min, siRNA transfection for 48 h. (PPTX 1031 kb) [file 12915_2020_749_MOESM4_ESM.pptx]

## Slide 1
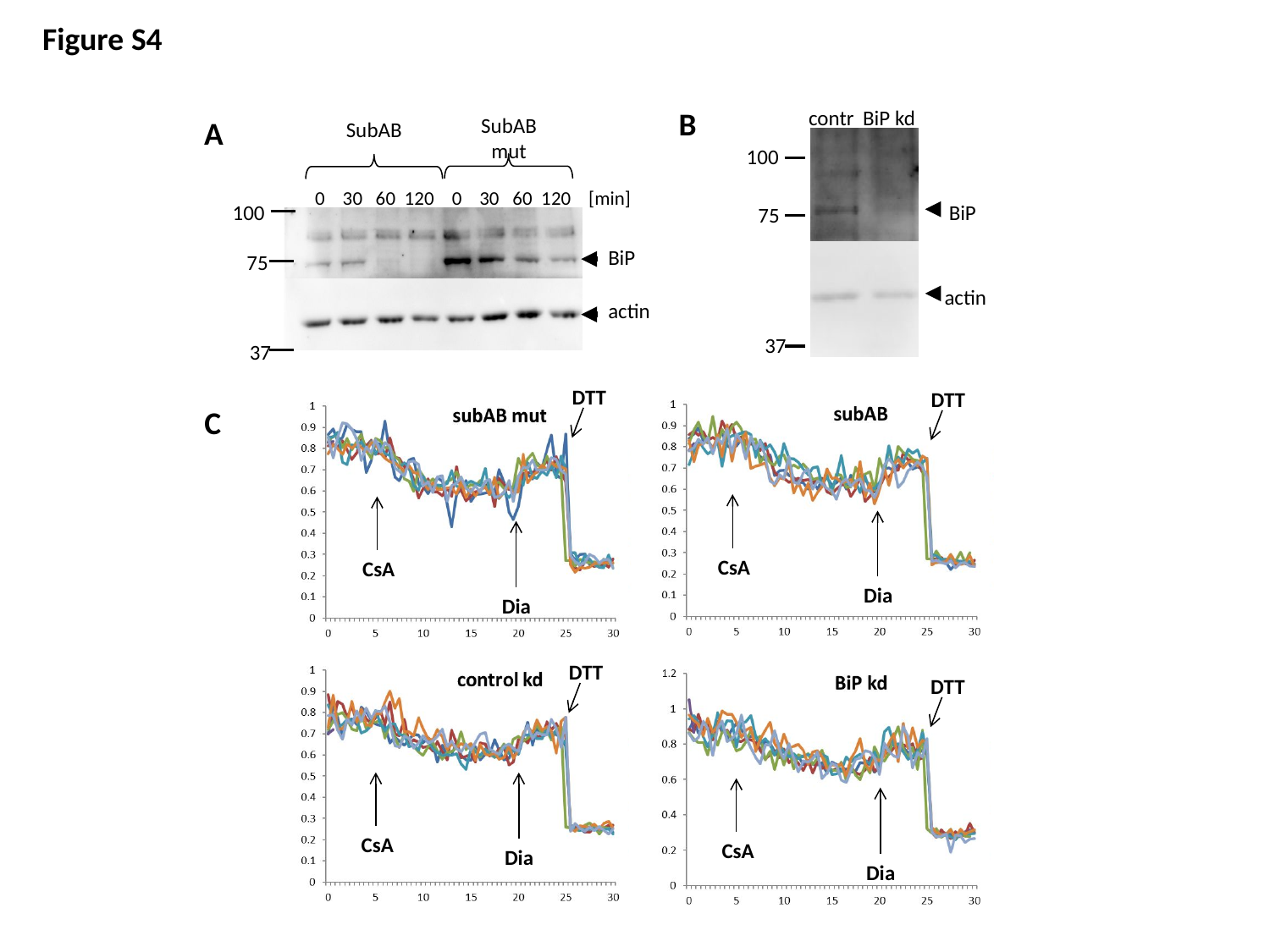

Figure S4
B
control
BiP kd
100
75
37
BiP
actin
SubAB mut
SubAB
100
BiP
75
actin
37
0 30 60 120 0 30 60 120 [min]
A
DTT
DTT
C
CsA
CsA
Dia
Dia
DTT
DTT
CsA
Dia
CsA
Dia
